# Esophagectomy in octogenarians: is it at a cost?^☆^

**DOI:** 10.1016/j.heliyon.2022.e11945

**Published:** 2022-11-28

**Authors:** Sujata Ojha, Muhammad B. Darwish, Annie L. Benzie, Shankar Logarajah, Patrick J. McLaren, Houssam Osman, Edward Cho, John Jay, D. Rohan Jeyarajah

**Affiliations:** a Department of Surgery, Methodist Richardson Medical Center, Richardson, Texas, USA; b Department of Surgery, TCU/UNTHSC School of Medicine, Fort Worth, Texas, USA; c Department of Surgery, The University of Oklahoma at Tulsa, Tulsa, OK, USA; d Department of Cardiothoracic Surgery, Methodist Dallas Medical Center, Dallas, Texas, USA

**Keywords:** Esophagectomy, Octogenarians, Geriatric surgery, Esophageal cancer

## Abstract

**Background:**

Surgical intervention in the geriatric population has a higher risk of perioperative morbidity and mortality due to frailty, comorbidities, and lack of compensatory physiologic reserve. The literature on esophagectomy in octogenarians is limited and there is concern about elderly patients being with-held surgery. The purpose of this study is to analyze the outcomes of esophagectomies for esophageal cancer in octogenarians to assess the safety of esophagectomy in this population.

**Methods:**

145 transhiatal esophagectomies performed for esophageal cancer between 2012 and 2020 were retrospectively reviewed in this IRB approved study. Two aborted esophagectomies were excluded. Patient demographics, surgical outcomes, and oncologic outcomes were reviewed. The octogenarian group was analyzed compared to patients younger than 80 years of age.

**Results:**

Among 143 esophagectomies, 136 patients were <80 years old while 7 were ≥80 years old. Octogenarians received significantly less neoadjuvant therapy compared to younger patients (42.9% vs 80.2%, p = 0.02). No statistically significant difference was noted in complication rate, length of stay (LOS), estimated blood loss (EBL), or mortality. However, octogenarians were found to have an increase in severity of complications compared to younger patients.

**Conclusion:**

This study demonstrates that esophagectomy can be performed in carefully selected octogenarians. This comes at a cost with increased severity of complications without an increase in complication rates or mortality. This data suggests that esophagectomy can be offered selectively to older patients with clear expectations and planning for the high risk of more severe post-operative complications.

## 1 Introduction

Esophageal cancer is a highly aggressive malignancy associated with a high mortality rate. It is the sixth leading cause of cancer-related death worldwide [1]. Esophageal cancer predominantly affects the elderly population and peaks in incidence after 65 years of age [2]. In 2021 the estimated number of new esophageal cancer cases is 19,260 followed by an estimated death of 15,530 individuals with a median age at diagnosis of 68 years [3].

Despite advancements in surgical techniques and perioperative therapy, esophageal cancer continues to have a poor prognosis with a 5-year relative survival rate of 19.9% [3]. Surgical intervention with an esophagectomy is the mainstay treatment for esophageal cancer and is a complex procedure that carries a high risk of morbidity and mortality [4]. As the population ages, it becomes increasingly important to understand the impact of esophagectomy in the elderly.

The literature on esophagectomy in octogenarians suggests that this procedure can be performed safely but there remains concern about older patients being denied surgery due to a bias regarding outcomes [5]. The current data available regarding this topic suggests that we should not write these patients off, but rather offer them a chance at surgery [6, 7, 8, 9]. Indeed, physiology, not chronology, should be the most important factor [10].

The purpose of this study is to analyze the surgical outcomes of esophagectomy for esophageal cancer in octogenarians to better assess the true risk these patients take by undergoing surgery. The unique aspect of this study, as opposed to others published, is that these surgeries were performed mainly using minimally invasive techniques and we asked the question if this might alter the outcome in this fragile group of patients. Moreover, the patients analyzed here underwent a transhiatal approach as opposed to the thoracic approach that has been studied by others [11, 12].

## 2 Methods

This study was approved by the IRB (037.HPB.2018.R) at Methodist Richardson Medical Center. A retrospective electronic chart review was undertaken of 145 consecutive transhiatal esophagectomies performed at a single institution for esophageal cancer from 2012 to 2020 using CPT codes (43107-8, 43280, 43286-9). Two aborted esophagectomies were excluded leaving 143 patients eligible for inclusion. Patient demographics, surgical outcomes, and oncologic outcomes were collected and analyzed. Patients were divided into two groups for comparison, those 80 years of age and older and those younger than 80 years. Reviewed surgical outcomes included complication rates, Clavien-Dindo (CD) score, 30-day mortality rates, average length of stay (LOS), and average estimated blood loss (EBL). All complications were classified based on the Clavien-Dindo classification system. Oncologic outcomes included TNM staging and resection margin status based on the American Joint Committee on Cancer (AJCC) 8^th^ edition guidelines. Groups were compared using nonparametric, univariate statistical analysis including Chi-square test, Fisher's exact test, and Mann-Whitney t-test. A p-value less than 0.05 was considered statistically significant. Statistical analysis was conducted using JASP (JASP Team (2020). JASP (Version 0.16.2) [Computer software]).

## 3 Results

Among 143 esophagectomies, 136 patients were <80 years old (range 39–79) and were 86.0% male while 7 were ≥80 years old (range 80–87) and 71.4% male. Most patients (80.2%) received neoadjuvant therapy in the <80 group and 42.9% of patients received NAT in the ≥80 group. Four of the patients in the ≥80 group did not receive NAT due to their clinical stage and one patient declined preoperative management. Thirty-three (24.3%) patients underwent open surgery in the <80 group compared to 2 patients (28.6%) in the ≥80 group. All other patients underwent minimally invasive transhiatal esophagectomy. There was no statistical difference in EBL between the octogenarians’ group (*M* = 1050 mL; SD = 1947.8 mL) compared to younger patients (*M* = 291.8 mL; SD = 324.1 mL) (Mann-Whitney U, p = 0.83). In addition, there was no statistical difference in the average LOS between octogenarians (*M* = 22.1 days; SD = 15.1 days) compared to younger patients (*M* = 11.6 days; SD = 7.1 days) (Mann-Whitney U, p = 0.06).

**Figure 1:**
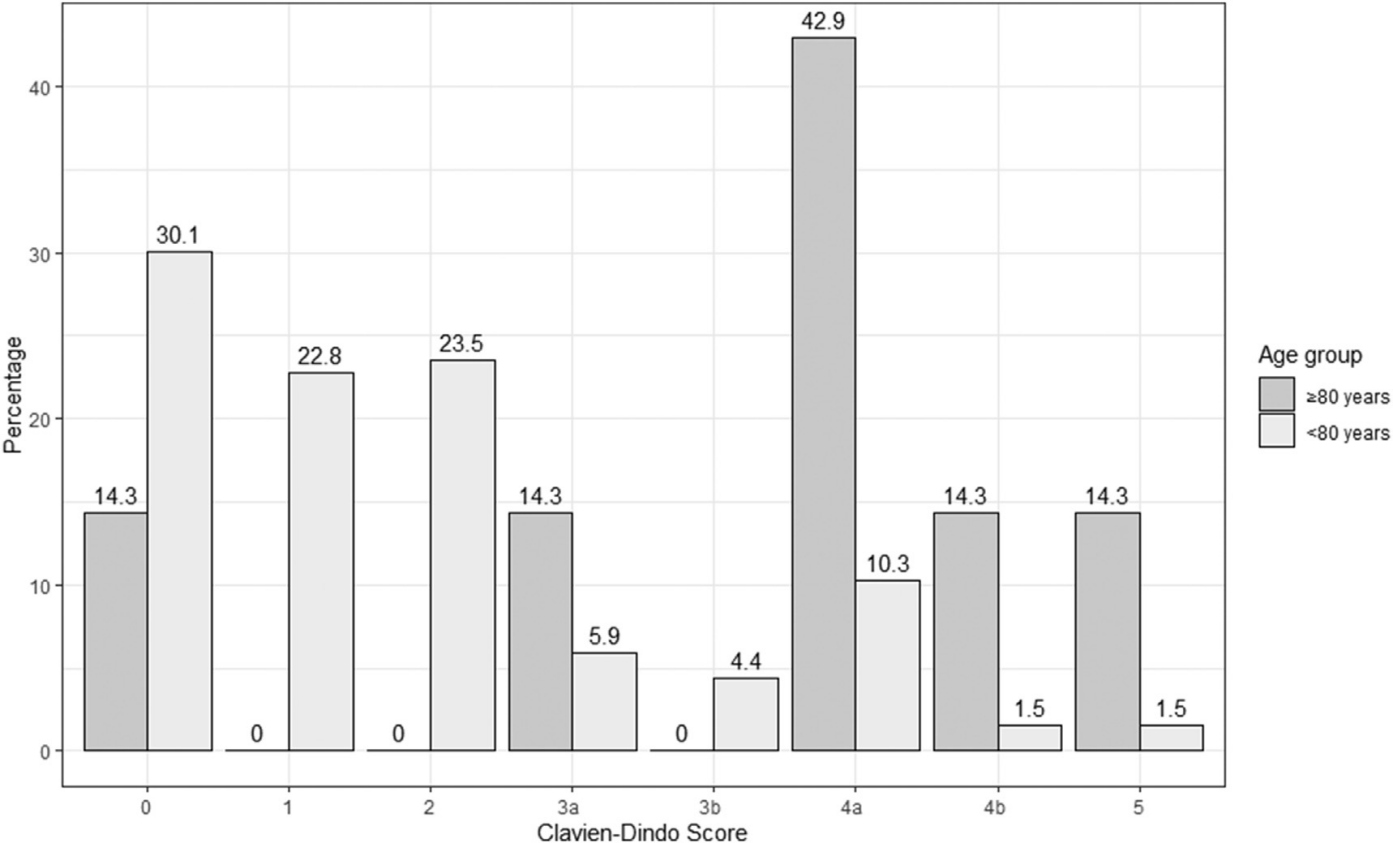
Clavien-Dindo grading for surgical complications.

The overall morbidity rate in octogenarians was 85.7% compared to 69.9% younger patients but did not reach statistical significance (X^2^ (1, N = 143) = 0.807, p = 0.369) [Table 1]. Complication were more severe in octogenarians as most complications were a CD 2 (23.5%) in the <80 group and a CD 4 (57.1%) in the ≥80 group (X^2^ (7, N = 143) = 21.2, p = < .01) [Figure 1]. The mortality rate demonstrated an upward trend in octogenarians (1/7; 14.3%) compared to younger patients (2/136; 1.5%) but did not reach statistical significance (p = 0.141) [Table 1].

**Table 1:** Surgery profiles.

	<80 years of age (n = 136)	≥80 years of age (n = 7)	p-value
Type of Surgery			*X*^2^ (1, N = 143) = 0.01, p = 0.79
Open	33 (24.3%)	2 (28.6%)	
MIE	103 (75.7%)	5 (71.4%)	
Average EBL (mL)	291.8	1050.00	*Mann-Whitney U*, p = 0.82
LOS (days)	11.6	22.1	*Mann-Whitney U*, p = 0.06
Average #LN∖	14.46	11.57	*Mann-Whitney U*, p = 0.29
Complication			*X*^2^ (1, N = 143) = 0.81, p = 0.46 0.37
Yes	95 (69.9%)	6 (85.7%)	
No	41 (30.1%)	1 (14.3%)	
Mortality	2 (1.5)	1 (14.3%)	*Fisher's exact test* p = 0.141

There were two mortalities in the <80 group. One patient had a history of hepatitis C and died on post-operative day 27 due to what appears to be hepato-pulmonary syndrome. The family elected to withdraw care. The second mortality occurred at day 11 in a patient with significant vascular disease and was thought to be due to bowel ischemia. This single mortality in the ≥80 group involved a patient who developed respiratory and cardiovascular complications on postoperative day 4 and the family elected to withdraw care.

Both adenocarcinoma and squamous cell histology were included in this study. Adenocarcinoma of the esophagus was the most common histologic type, with a total of 118 cases, accounting for 113 patients in the <80 category (83.1%) and 5 in the ≥80 category (71.4%).

Resections margins were negative for 115 (84.6%) patients in the <80 group and 6 (85.7%) patients in the ≥80 age group [Table 2]. It is worth noting that MIE resulted in significantly higher rates of negative resection margins (89.8%) compared to OE (68.6%, p = < .01).

**Table 2:** Pathologic profiles.

	<80 years of age (n = 136)	≥80 years of age (n = 7)
pT (*X*^*2*^ (1, N = 143) = 3.61, p = 0.82)		
0	19 (14.0%)	1 (14.3%)
1	3 (2.2%)	0
1A	16 (11.8%)	2 (28.6%)
1B	24 (17.6%)	2 (28.6%)
2	14 (10.3%)	1 (14.3%)
3	55 (40.4%)	1 (14.3%)
4A	1 (0.74%)	0
Tis	4 (2.9%)	0
pN (*X*^*2*^ (1, N = 143) = 3.54, p = 0.47)		
0	82 (60.3%)	4 (57.1%)
1	25 (18.4%)	3 (42.9%)
2	18 (13.2%)	0
3	10 (7.4%)	0
X	1 (0.74%)	0
Grade (*X*^*2*^ (1, N = 143) = 3.24, p = 0.66)		
0	1 (0.74%)	0
1	10 (7.4%)	1 (14.3%)
2	59 (43.4%)	1 (14.3%)
3	43 (31.6%)	4 (57.1%)
4	2 (1.5%)	0
X	21 (15.4%)	1 (14.3%)
Resection Margins		
- Open		
o Negative	23 (69.7%)	1 (50.0%)
o Positive	10 (30.3%)	1 (50.0%)
- MIE		
o Negative	92 (89.3%)	5 (100.0%)
o Positive	11 (10.7%)	0 (0.0%)
- Total (*X*^*2*^ (1, N = 143) = 9.16, p = < .01)		
o Negative	115 (84.6%)	6 (85.7%)
o Positive	21 (15.4%)	1 (14.3%)
Pathology		
- Adenocarcinoma	113 (83.1%)	5 (71.4%)
- Squamous Cell Carcinoma	18 (13.2%)	1 (14.3%)
- Neuroendocrine tumor	3 (2.2%)	0 (0.0%)
- Adenoneuroendocrine tumor	1 (0.7%)	0 (0.0%)

## 4 Discussion

This study shows that esophagectomy in octogenarians for esophageal cancer comes at a cost with an increased severity of post-operative complications but no significant difference in complications or mortality rates. This study highlights the importance of carefully selecting octogenarians for esophagectomy with a clear understanding that the risk of more severe post-operative complications is higher. Compared to the alternative, the authors concede that esophagectomy should be offered to the octogenarian.

The aging population of the United States and the aging baby boomer population will cause a demographic shift where the number of older people will outnumber children for the first time in U.S. history [13]. It is expected that by the year 2040, 14.4 million people will be older than 85 years of age, more than doubling the population over the age of 85 in 2020 [14]. The increasing age will lead to increasing incidence of cancer and subsequently increase the number of older people needing surgical treatment.

Several studies have analyzed the outcomes of esophagectomy in octogenarians with esophageal cancer with some showing an increase in complications in the elderly population [7, 15, 16] while others showed no difference when compared to a younger cohort [8, 17, 18]. Markar et al. conducted an analysis looking at the outcomes of 500 esophagectomies, of which 32 were octogenarians [7]. The study reported that patients who were ≥80 years had a greater incidence of postoperative morbidity [7]. The authors hypothesize that this was due to underlying comorbidities and a low rate of NAT [7].

A ten-year prospective cohort study looking at 1,777 patients who underwent an esophagectomy reported a postoperative morbidity rate of around 50% and thirty-day mortality rate of 10% [16]. The authors extrapolate that perioperative factors including increasing age, preexisting comorbidities like diabetes mellitus and chronic obstructive pulmonary disease were risk factors for increased overall morbidity [16]. Furthermore, intraoperative variables such as a need for intraoperative blood transfusion, increased time spent in the operating room, and emergency status of the patient predicted an increase in morbidity [16]. In fact, our results would suggest that the differences between the ≥80 and <80 groups in this study may be explained by the very low mortality in the younger group. In fact, the mortality in the octogenarian group is comparable to studies of all esophagectomies, making the argument even stronger for surgery in this group in our hands [16, 19, 20].

In a study by Tapias et al analyzing the short and long-term outcomes after esophagectomy for elderly patients, a significant increase in postoperative major complications (62.5%) in octogenarians was noted compared to patients aged 70 to 79 years (47.6%) and patients under the age of 70 years (37.2%) [21]. Other studies have also shown an increase in mortality for octogenarians undergoing esophagectomy [7, 15]. Our study showed an increase in complication severity, as demonstrated by the CD score. However, while mortality in the octogenarian group demonstrated an upward trend, we did not observe a statistical significance.

Optimizing patients for surgery with preoperative rehabilitation, maximizing nutritional status, and NAT are crucial in reducing the risk of postoperative morbidity [22, 23, 24]. The treatment of esophageal cancer presents a challenge when considering NAT for elderly patients. The decision to offer therapy is determined by carefully weighing the risk of toxicity, risks and benefits of subsequent surgery, and life expectancy [25]. Octogenarians tend to receive less NAT compared to younger patients due to their advanced age [26]. While NAT has been shown to improve the rate of complete resection when combined with surgery, surgery alone remains the single most important factor in increasing survival in esophageal cancer [27]. In this study, a trend towards a higher positive resection margin rate was observed with open esophagectomy compared to minimally invasive esophagectomy. The authors hypothesize that this is likely related to the overall higher pT stage in patients undergoing open esophagectomy compared to MIE.

Minimally Invasive Esophagectomy (MIE) has been compared to Open Esophagectomy (OE) in several systematic literature reviews [28, 29]. Some studies show an improvement in perioperative morbidity with MIE compared to OE [30, 31]. Offering octogenarians an MIE can decrease their post-operative morbidity and improve their oncologic outcomes including resection margin status [32].

It is important to note the weaknesses of this study. This is a retrospective study analyzing data from a single practice of three surgeons. The sample size is small and disproportionate which makes it difficult to make a generalizable statement and underpowers the statistical analysis. The study looked at patients that underwent an esophagectomy for esophageal cancer but did not review patients that were withheld surgery for medical or oncologic reasons. Data on octogenarians with esophageal cancer that elected not to undergo surgery was not available which presents a selection bias. Lastly, there were two aborted cases due to significant intra-operative bleeding and unresectable tumor, respectively. These two cases were in the <80 years of age group and present a potential bias in the analysis. Further research with a larger sample size is warranted.

## 5 Conclusion

This study demonstrates that esophagectomy can be performed in carefully selected octogenarians. This comes at a cost with increased severity of complications without an increase in complication rates. This data suggests that esophagectomy can be offered selectively to older patients with clear expectations and planning for the high risk of more severe post-operative complications.

## Declarations

### Author contribution statement

Sujata Ojha, Muhammad B. Darwish and D. Rohan Jeyarajah: Conceived and designed the experiments; Analyzed and interpreted the data; Wrote the paper.

Annie L. Benzie, Shankar Logarajah, Patrick J. McLaren, Edward Cho, Houssam Osman and John Jay: Analyzed and interpreted the data; Wrote the paper.

### Funding statement

This research did not receive any specific grant from funding agencies in the public, commercial, or not-for-profit sectors.

### Data availability statement

Data will be made available on request.

### Declaration of interest's statement

The authors declare no conflict of interest.

### Additional information

No additional information is available for this paper.
